# A different kind of weapon focus: simulated training with ballistic weapons reduces change blindness

**DOI:** 10.1186/s41235-016-0037-0

**Published:** 2017-01-30

**Authors:** J. Eric T. Taylor, Jessica K. Witt, Jay Pratt

**Affiliations:** 1 Brain and Mind Institute, The University of Western Ontario, 1151 Richmond St N, London, Ontario N6A 5B7 Canada; 2 Colorado State University, Fort Collins, CO USA; 3 University of Toronto, Ontario, Toronto Canada

**Keywords:** Attention, Change blindness, Weapon training, Action and attention

## Abstract

Attentional allocation is flexibly altered by action-related priorities. Given that tools – and specifically weapons – can affect attentional allocation, we asked whether training with a weapon or holding a weapon during search would affect change detection. In three experiments, participants searched for changes to agents, shootable objects, or environments in the popular flicker paradigm. Participants trained with a simulated weapon or watched a video from the same training perspective and then searched for changes while holding a weapon or a control object. Results show an effect of training, highlighting the importance of sensorimotor experience for the action-relevant allocation of attention, and a possible interaction between training and the object held during search. Simulated training with ballistic weapons reduces change blindness. This result has implications for the interaction between tool use and attentional allocation.

## Significance

Change detection is essential to policing work, hunting, military sentry, or any other activity where a threat or target might demand action with a ballistic weapon. In these types of tasks, the observer is typically required to surveil a scene vigilantly, searching for changes. Change blindness is therefore a liability and the efficient detection of changes could increase the time available to assess threat – thereby reducing errors in categorization that can tragically lead to mistakes. Given the importance of change detection to gun-related activities and the emerging link between tool use and attentional allocation, we asked whether training with a gun or holding a gun would affect change detection.

Change detection is essential to tasks of vigilance or surveillance. Sometimes, as in the case of police work or other sentry duties, the motivation for vigilance may be detecting threat, which can be acted upon with ballistic weapons. Sadly, the misallocation of visual attention in these scenarios can have dangerous consequences, such as when a new stimulus is incorrectly identified as a weapon in the hands of a potential threat. Accordingly, we asked whether the actions afforded by ballistic weapons influence attentional allocation while searching for changes. We manipulated participant experience with a weapon during training and search in the popular flicker paradigm. To preview our results, we found limited evidence for the effect of weapon affordances on change detection, but strong evidence for the role of training. In other words, sensorimotor experience reduced change blindness, compared to visual experience alone.

Change detection requires focal attention at the location of a changing stimulus. This is demonstrated using the flicker paradigm, where two slightly different images alternate with an interleaved mask until response. Participants are tasked with identifying the difference between the images. Differences are easier to spot when they are cued, supporting a role for attention (Rensink, O’Regan, & Clark, 1997). Because change detection requires attention, the flicker paradigm is a useful tool to discern an observer’s attentional biases. For example, alcohol, cannabis, and cigarette users are faster to detect changes to the stimulus corresponding to their habit (Jones, Jones, Smith, & Copley, 2003; Yaxley & Zwaan, 2005), because they attend to these stimuli over other elements in the display. Thus, performance on a flicker task reveals biases in attentional allocation.

Much research on attentional allocation focuses on characteristics of the optical stimulation. However, the allocation of attention can also be flexibly modulated by action. For example, on-path distractors capture attention when reaching to a target compared to equidistant off-path distractors, showing how attention prioritizes stimuli in reach-relevant space (Pratt & Abrams, 1994; Tipper, Lortie, & Baylis, 1992) and visual search for graspable objects reduces the number saccades to wrong-orientation distractors when grasping (Bekkering & Neggers, 2002). These action-relevant attentional priorities also translate to change detection, as observers making a precision or a power grasp response are faster to detect changes to grasp-congruent objects (Symes, Tucker, Ellis, Vainio, & Ottoboni, 2008) and change detection for orientation is better when preparing a grasp versus a pointing action (Gutteling, Kenemans, & Neggers, 2011). Together, these studies show how planning and executing actions flexibly alters the priorities of selective attention, which can be revealed using change detection tasks.

Using tools rapidly changes action affordances, causing corresponding changes to perception (Witt, Proffitt, & Epstein, 2005) and attentional allocation (Reed, Betz, Garza, & Roberts, 2010; Taylor & Witt, 2014). In the case of ballistic weapons, attentional allocation (or misallocation) can have deadly ramifications. Consequently, it is prudent to examine how using and training with weapons alters attentional allocation. Recent research shows that holding a gun increases the bias to categorize other objects as guns (Witt & Brockmole, 2012) and that holding a gun increases dwell time for faces during free viewing (Biggs, Brockmole, & Witt, 2013), but it remains unknown whether holding weapons changes the way we scan a scene for changes. Given the importance of change detection to real-world policing and military behaviors, we examined whether training and/or holding a weapon during search would alter the allocation of attention in the flicker paradigm.

## Experiment 1

To assess the effect of weapon affordances on attentional allocation, we trained observers in a video game for which they used a gun-shaped implement to destroy various stimuli. Later, they grasped the weapon or a ball as they searched for changes in a flicker task with stimuli drawn from that game. Therefore, the training session involved playing a game in a dynamically changing environment and the search session involved scanning still images previously taken and altered from the same game. Changes occurred to agents (e.g. spiders, monsters, etc.), destructible objects (e.g. pots, boxes, targets), or inanimate features of environments (e.g. buildings or mountains). We asked whether holding the weapon attenuated change blindness and whether this weapon-based attenuation effect would interact with the type of change, as agents and objects provide an affordance for shooting, whereas environments do not.

### Method

#### Participants

Twenty students participated for course credit (11 women; mean age = 19.65 years; *SD* = 1.53).

#### Materials

**Fig. 1:**
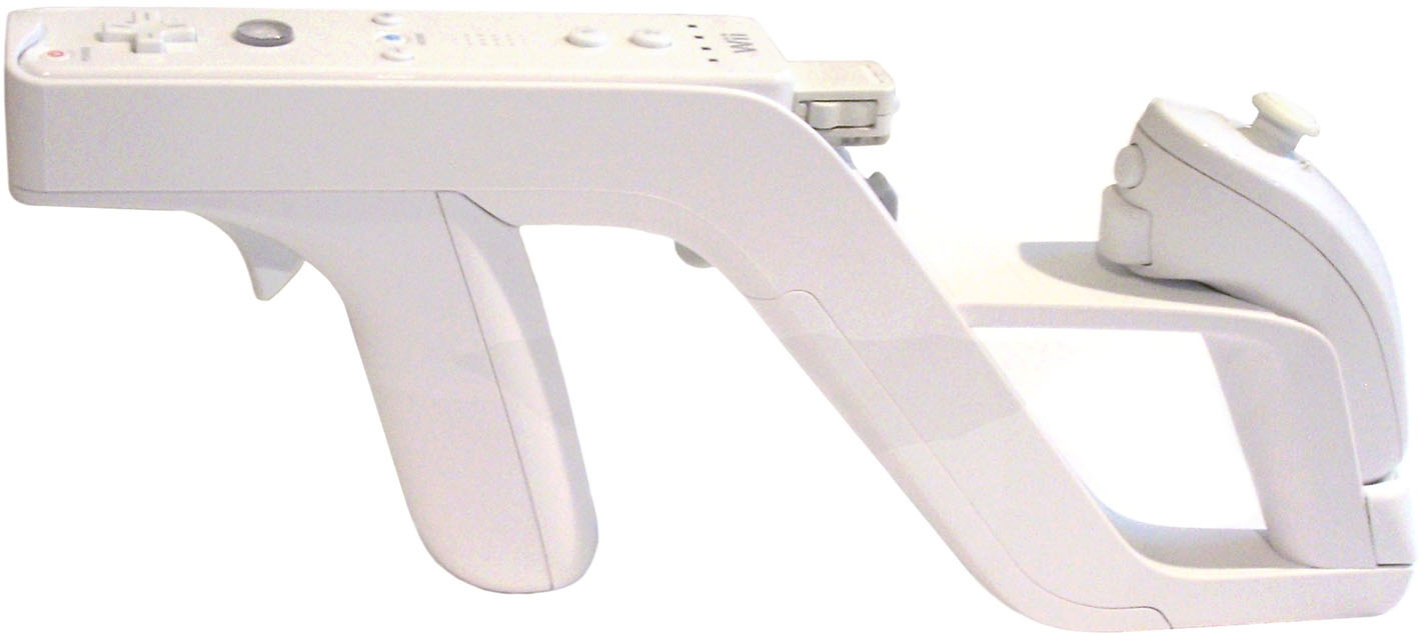
The Wii Zapper. Image from Wikimedia

All stimuli were taken from *Link’s Crossbow Training* for the Nintendo Wii. During training, participants played through six pre-selected levels that introduced the player to a wide range of environments, agents, and destructible objects. The Wii console uses an infrared sensor placed near the display to detect infrared light emitted by the controller. Consequently, it tracks movement of the controller, allowing the player to interact with stimuli on the display with high spatial and temporal resolution. In this experiment, the controller is inserted into the *Wii Zapper*, which is gun-shaped with a trigger and is held in a two-handed posture like a rifle (Fig. 1). The player plays by pointing and shooting at enemies and objects on the display. After a fixed amount of time, the perspective moves. Because the game is of the rail shooter genre, the player’s perspective is fixed, but they have the freedom to look around, aim, and shoot. Thus, differences in visual experience between participants in the training phase can be attributed entirely to which things they do or do not shoot and where they direct the reticle of their weapon. Critically, participants all saw the same agents, objects, and environments.

At test, participants viewed images from the game they had just played. These images were screen-grabbed and altered using Photoshop. The alterations were always the presence or absence of an element: either an agent, object, or a feature of the environment. There were ten unique image sets for each category, for a total of 30 test stimuli. Each type of change was presented equally often in different settings. For the forest environment, for example, the change was equally likely to be an agent, object, or environment. All participants saw the same 30 image sets.

The training game and the stimuli for the test phase were displayed on a projector facing a white wall in a dark room. Participants stood behind the projector, where they completed both the training and test phase.

#### Procedure

During training, all participants played six levels of the game, each lasting between 1 and 2 min. The duration of each level was fixed, controlling for exposure to the training stimulus. Participants stood behind the projector, using the weapon to point and shoot at items on the display. In three of the six levels, the task was to shoot objects such as barrels or targets. In the other three levels, their task was to shoot threatening agents that would approach them on the display. This ensured that participants had experience shooting agents and objects.

**Fig. 2:**
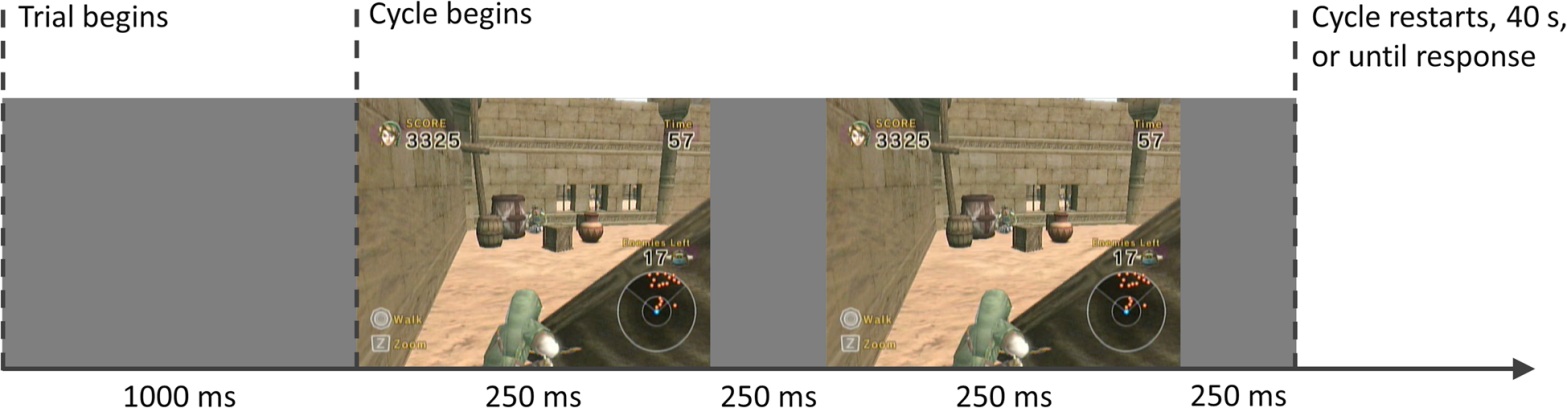
Example trial. Here, an additional window appears on the wall – an environmental change. See text for details

Participants were assigned to one of two search groups for the test phase: holding the same weapon with which they had trained, or a hand-sized ball. Participants were assigned to each group in alternating order. Participants initiated a trial by pressing and holding a large button on the table in front of them with their object (weapon or ball). After 1000 ms, a source image and a modified image were presented in alternating order for 250 ms with interleaved gray masks (Fig. 2). Participants searched the image for a change or until 40 s had passed, at which point the trial was terminated. Upon detecting a change, they raised their tool and pointed it at the changed item. Releasing the button ended the trial, measuring reaction time (RT), and ending stimulus presentation. Participants were then required to name the change. The experimenter input the response on a separate monitor not visible to the participant. No feedback was given. Participants knew the change would occur to an agent, object, or environment and that the change would be the alternating presence and absence of an item. Participants were instructed to react as quickly as possible while minimizing errors. The 30 flicker stimuli were presented in a randomized order. The experiment was 2 (search: weapon or ball; between-subjects) × 3 (change: agent, object, or environment; within-subjects) mixed-factors design.

### Results and discussion

**Fig. 3:**
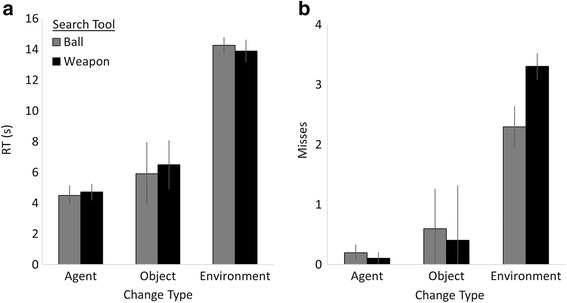
Mean reaction times (**a**) and number of errors (**b**) for Experiment 1. *Error bars* represent one standard error of the mean

Responses for which the participant failed to identify the change before the trial timed out (40 s) or incorrectly identified the change were categorized as errors. Mean RTs for correctly-identified trials for each participant were entered into a 2 (search tool: weapon or ball; between-subjects) × 3 (change stimulus: agent, object, or environment) ANOVA. One participant’s data could not be entered into this ANOVA because they had no successful detections in one cell and thus was excluded from further analyses. Change type significantly influenced RT, *F*(2,34) = 49.58, *p* < 0.001, η_p_^2^ = 0.74 (Fig. 3). Participants were significantly faster in detecting changes to agents than to objects or the environment (*t*s > 3.65, *ps* < 0.002), replicating the finding that attention prioritizes animate things (New, Cosmides, & Tooby, 2007).^1^ Importantly, for the purpose of our study, there was no effect of search tool and no interaction, *F*s < 0.11, *p*s > 0.897.

The mean number of errors was examined with the same ANOVA. Errors include misses and false alarms. There was a significant main effect of change type, *F*(2,36) = 22.38, *p* < 0.001, η_p_^2^ = 0.55. Because participants were both faster and more accurate for changes to animate objects, we can discount the possibility of a speed-accuracy trade-off. No other effects reached significance, *F*s < 1.20, *p*s > 0.314.

We expected that participants would rapidly adapt to the actions afforded by the weapon and that holding that weapon during search would increase the likelihood of detecting shootable things. Instead, we found that it made no difference whether they searched holding the weapon or an inert ball. It is possible that the training phase did alter their allocation of attention and that they simply simulated those actions in both the weapon and ball conditions during the test phase. Alternately, it is possible that training with a weapon had no effect on attention during search, where the weapon was inert. In other words, weapon training may only change the allocation of attention while holding a weapon that is “on.” To assess these possibilities, we ran a second experiment, with fully crossed training and test phases.

## Experiment 2

To assess interactions between training and holding a weapon during change detection, we had participants either play the game with the weapon (as in Experiment 1) or watch a screen-grabbed video of someone experiencing the training session while holding a ball. Critically, this video depicted the exact same levels from the same perspective that the participants in the weapon training condition experienced, including changes due to locomotion. Consequently, the visual exposure in the weapon and ball training conditions was highly similar; the difference between conditions lies in the sensorimotor experience of using the weapon for action versus holding a ball, watching passively.

### Method

#### Participants

Forty-eight students participated for course credit (21 women; mean age = 19.79 years; *SD* = 2.14).

#### Materials and procedure

The materials and procedure were identical to Experiment 1 except that there was an additional between-subjects factor of training. In Experiment 2, half of the participants trained in the game with the weapon, using it to destroy on-screen elements, while the other half held a ball and watched a screen-captured video of a player in the same levels. The video in the ball training condition depicted the same perspective, environments, and adversaries as the levels experienced by the weapon training condition. Thus, Experiment 2 was a 3 (change: agent, environment, or object; within-subjects) × 2 (training: weapon or ball; between-subjects) × 2 (search: weapon or ball; between-subjects) design. Twelve participants participated in each of the four combinations of tool training and tool use.

### Results and discussion

**Fig. 4:**
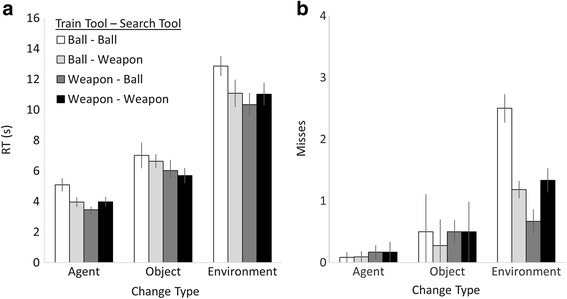
Mean reaction times (**a**) and number of errors (**b**) for Experiment 2. *Error bars* represent one standard error of the mean

Responses for which the participant failed to identify the change before the trial timed out (40 s) or incorrectly identified the change were categorized as errors. One participant was removed prior to analysis for being hungover. Mean RTs for correctly identified trials for each participant were entered into a 2 (training: weapon or ball; between-subjects) × 2 (search: weapon or ball; between-subjects) × 3 (change: agent, object, or environment) ANOVA. There was a main effect of change type, *F*(2,86) = 158.24, *p* < 0.001, η_p_^2^ = 0.79 (Fig. 4), replicating the agent-specific benefit over objects and environments (*t*s > 6.75, *ps* < 0.001). Critically, there was a main effect of training tool, *F*(1,43) = 7.35, *p* = 0.010, η_p_^2^ = 0.15. Search times were faster for those who had trained with the gun, actively interacting with the display, compared with those who had searched while holding a ball. Moreover, the interaction between training tool and search tool was approaching significance, *F*(1,43) = 3.43, *p* = 0.071, η_p_^2^ = 0.07. No other main effects or interactions reached significance, *F*s < 1.14, *p*s > 0.292.

The mean number of errors was examined with the same ANOVA. There was a significant main effect of change type, *F*(2,86) = 28.71, *p* < 0.001, η_p_^2^ = 0.40, again showing that there was not a speed-accuracy trade-off. There was an interaction between change type and training tool, *F*(2,86) = 3.63, *p* = 0.03, η_p_^2^ = 0.08, which was qualified by a three-way interaction, *F*(2,86) = 3.67, *p* = 0.03, η_p_^2^ = 0.08, which is caused by more errors to environmental changes in the ball–ball condition. We suspect this interaction is largely spurious, given the small number of errors overall.

Participants who played the game with an active weapon were more sensitive to changes during a later search even though neither search tool (weapon nor ball) was actually functional during search. One explanation for this finding is that the ability to destroy elements during training was later simulated during search, regardless of which tool was being held. This weapon simulation enhanced the allocation of attention across the entire display. Interestingly, this effect did not interact with change type, suggesting that the attentional improvement was uniform rather than specific the destructible items. And although it should be interpreted with caution due to its marginal significance, there was some evidence of an interaction between training tool and search tool, as participants who watched the videos and searched with the ball were slower than groups who had experience with the weapon.

## Experiment 3

There are two alternative accounts for the data from the first two experiments. First, it is possible that the observed effect of training with the tool is a product of elevated arousal, rather than sensorimotor training. By this account, playing the game is more exciting than watching videos of the same scenes and the elevated arousal might increase attention to threats, reducing attention to peripheral stimuli and reducing change blindness. This logic is akin to the original weapon focus literature (Fawcett, Russell, Peace, & Christie, 2013). To test this alternative, we required a training condition that does not involve the same postures or actions as holding the simulated weapon, but is equally arousing. To this end, we collected data in a condition where participants trained on the game using a controller that required an awkward posture and was not weapon-like.

Second, the object that is held during training is confounded with the training itself; participants held a ball and watched or held a simulated weapon and played. To resolve this confound, we collected data in a condition where participants held the weapon-like controller and watched the videos passively.

We compared both new conditions against a replicated condition of the weapon–weapon condition of Experiment 2, with the expectation that this active weapon condition should produce faster change detection times than the other conditions.

### Method

#### Participants

Thirty-three students participated for course credit or financial compensation (26 women; mean age = 21.21 years; *SD* = 3.16).

#### Materials and procedure

The method was identical to Experiments 1 and 2 with the following exceptions. The experiment was conducted at a different institution, on a large flat-screen monitor instead of a projector. In the active weapon condition, participants played the game with the weapon, then conducted the search holding the weapon (replication of weapon–weapon condition from Experiments 1 and 2). In the passive weapon condition, participants held the weapon and watched the screen-grabbed videos, then searched while holding the weapon. In the Wiimote condition, participants played the game during training but used a different, non-weapon controller. The standard Wiimote controller is an elongated, rectangular prism that players point at the screen. Connected to it by a long wire is a smaller “nunchaku” controller with a joystick. Players were instructed to play the game with the wand and to hold the nunchaku behind their back. They searched the display with the same wand controller in hand.

### Results and discussion

**Fig. 5:**
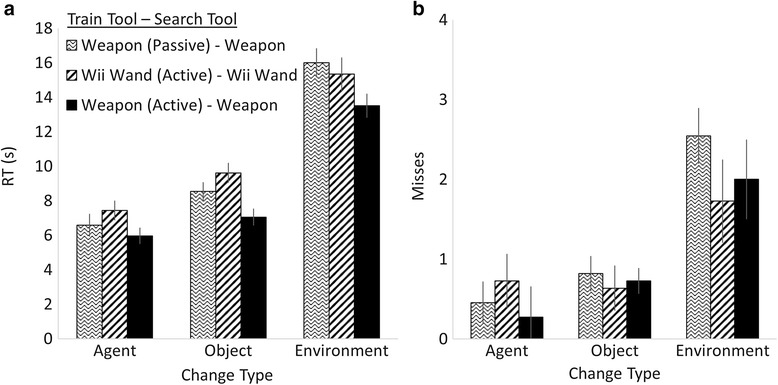
Mean reaction times (**a**) and number of errors (**b**) for Experiment 3. *Error bars* represent one standard error of the mean

Responses for which the participant failed to identify the change before the trial timed out (40 s) or incorrectly identified the change were categorized as errors. Unlike Experiments 1 and 2, the training and search factors were not fully factorial, so treating the three conditions as a between-subjects factor is appropriate. Moreover, the lack of training and search interactions with change type motivated us to focus on the comparison between the weapon–weapon condition and the two control conditions.^2^ Consequently, mean RTs for correctly identified changes were entered into a between-subjects ANOVA contrast between the weapon–weapon condition and the other two conditions, where we observed a significant effect, *F*(1,31) = 5.79, *p* = 0.022, confirming that participants in the weapon–weapon condition detected changes faster than the controls (Fig. 5). The same contrast was conducted on the mean number of errors; there was no effect (*F* < 1).

Participants who played the game detected changes faster than participants who held onto the simulated weapon and watched the game or participants who played the game with a controller that used a non-weapon posture. These results resolve the confounding of training object and training task in Experiment 2 and, in addition to replicating our result, they rule out the alternative explanation that the present training effects are caused by general arousal or engagement with the game.

### General discussion

Across three experiments, participants trained and searched for changes while holding a weapon or a ball. During training, participants using the simulated weapon could actively destroy on-screen elements, whereas participants using the ball could not. We found that participants who had trained with the weapon were later better able to detect changes to stimuli regardless of the object that changed (agent, object, or feature of the environment). This result shows that vigilant surveillance benefits from sensorimotor training with the tool – in this case, a simulated weapon – that you would use to interact with the environment.

Another important finding is the null effect of the search tool. It made no difference whether observers searched with the weapon or ball. This violated our expectations, given that action affordances flexibly alter attentional allocation during change detection (Symes et al., 2008) and that tool use in general – and guns specifically – alter attentional location (Biggs et al., 2013). Accordingly, we predicted that holding the weapon during search would confer advantages, or at least produce biases, compared with holding the ball.

We can, however, cautiously interpret a marginal interaction between the training tool and the search tool in Experiment 2. Participants who trained and searched with the ball were slowest to detect changes across the board, whereas participants who trained with the ball (no sensorimotor training; only visual) and searched with the gun expressed search latencies more like the groups who trained with the gun. It appears that any exposure to the weapon, whether during training or search, was sufficient to attenuate change blindness. The tantalizing implication drawn from this result is that participants who trained with the ball but searched with the weapon were as capable of simulating the weapon-related action affordances during search as those who trained with the weapon.

Interestingly, experience with the weapon led to an item-non-specific attenuation of change blindness. In other words, changes of all types were detected faster after training with the weapon. If the observed attenuation of change blindness is indeed caused by variations in attentional allocation resulting from simulated weapon affordances, then we might expect that shootable elements – agents and objects – should be prioritized during search over environmental elements in the weapon conditions. Instead, we found that simulated weapon training led to a blanket improvement in change detection times regardless of change type, suggesting that change detection is universally improved by sensorimotor training. This finding sets the current study apart from existing research on domain-specific expertise and change blindness. Experts in solving physics problems (Feil & Mestre, 2010) or watching football (Werner & Thies, 2000) are faster at detecting changes to domain-relevant images as compared to novices. However, these studies examine true expertise derived from perceptual experience that goes well beyond the ~10 min practiced by our participants. Moreover, the item-non-specific attenuation of change blindness we observed sets the present findings apart from domain-relevant attenuation of change blindness. A domain-relevant effect in the present study would occur only for shootable things (agents and objects). We found no interaction between training tool and change category, indicating that the attenuation of change blindness was not domain-relevant as it is in existing investigations of perceptual expertise.

Additionally, the effect of change type, wherein changes to agents were detected faster than inanimate elements, draws comparisons to the animate monitoring hypothesis (New et al., 2007). It is not surprising that agents should be detected fastest, especially considering that they were all threatening stimuli in the present study, however it should be noted that destructible objects were detected much faster than environmental elements. In previous studies examining animacy and change detection, artifacts and topographical landmarks are detected equally quickly (New et al., 2007). In contrast, we found a large advantage for objects over environments. We propose that objects’ affordance for destruction led to improved attentional allocation over the environmental changes.

In many real-world scenarios, the critical change – for example, the sudden appearance of a threat – occurs only once, rather than alternating as in the flicker paradigm. For this reason, it would be helpful to conduct future investigations using the one-shot change detection paradigm, where an image is presented for a short duration, followed by an intervening mask and a slightly changed image (e.g. Phillips, 1974). We used the flicker paradigm, where the images alternate repeatedly, because we reasoned the potential for weapon use would occur in scenarios where the observer was engaged in prolonged surveillance rather than a brief exposure. Both methods provide unique insights into the nature of change detection (Rensink, 2002) and the applied question of change detection during vigilant surveillance. For example, the flicker paradigm is better suited for measuring the speed of change detection, whereas the one-shot paradigm typically measures accuracy.

Experiment 3 was conducted with the intent of isolating the best control conditions to compare against the weapon–weapon training-search condition. Because of these comparisons, we can confidently attribute our finding to sensorimotor training with a simulated weapon: change blindness was attenuated following simulated training with a weapon compared to visual-only and arousal-matched control conditions. Another interesting comparison would have been a no-training condition, where participants had no exposure to the stimuli at all prior to the change detection task. The marginal interaction between training and search conditions in Experiment 2 suggests it is possible that sensorimotor training is not required to attain this improved change detection; searching with the simulated weapon may be sufficient. Consequently, it may be possible that an observer with no prior exposure at all to the stimuli could achieve a similar improvement in change detection just by holding a weapon. This speculative result imagines a strong weapon affordance effect on attention.

In the real world, training regimens for police and military duties extend far beyond the ~10 min practiced by our participants. Because the training period was short, and because there was no delay between training and search, we can only speak of short-term effects on vision. The effect of simulated weapon training can be attributed to sensorimotor experience, but it is possible that long-term training effects can be elicited with only sensory training. More research is necessary to determine whether sensory-only training can produce long-term improvements in attentional allocation in sentry-type tasks. The restriction of our conclusions to the short term reveals an additional point of interest: that we observed attenuated change blindness as a result of simulated weapon training indicates that these types of training affordances incur immediate effects on attentional allocation, consistent with a rapid integration of tool affordances into the body schema. This is concordant with some of our earlier research showing that brief exposure to a new tool stimulus is sufficient to alter the allocation of attention to and near that tool (Taylor & Witt, 2014, Experiment 4; also Reed et al., 2010).

In conclusion, training with a simulated weapon led to immediate item-non-specific attenuation of change blindness. This effect reveals the importance of training regimens when it comes to the use of weapons and more generally suggests that sensorimotor experience with a tool is essential to activate action-related attentional priorities.

## Footnotes

1 It is possible that low-level visual factors besides the changes’ semantic category influenced this pattern. We measured the mean size and distance from the center of all changes. In a one-way ANOVA, there was no effect of change category on distance from the center. There was an effect of mean change size, *F*(2,27) = 7.72, with environmental changes being the largest by far; comparing agent and object size yielded no effect, *t*(18) = 1.25, *p* = 0.227. These results refute the possibility that differences in size or distance caused agents to be prioritized.

2 A supplementary analysis confirmed there was, again, an effect of change type (*p* < 0.001) but no interactions with the between-subjects factor (*F* < 1).
